# Effects of Supra-Physiological Levothyroxine Dosages on Liver Parameters, Lipids and Lipoproteins in Healthy Volunteers: A Randomized Controlled Crossover Study

**DOI:** 10.1038/s41598-017-14526-2

**Published:** 2017-10-26

**Authors:** Barbara Sjouke, Laura P. B. Elbers, Bregje van Zaane, John J. P. Kastelein, G. Kees Hovingh, Victor E. A. Gerdes

**Affiliations:** 1 Department of Vascular Medicine, Academic Medical Center, PO box 22660, 1100 DD Amsterdam, The Netherlands; 2 Department of Internal Medicine, Slotervaart Hospital, PO box 90440, 1006 BK Amsterdam, The Netherlands

## Abstract

Eprotirome, a liver specific thyroid hormone agonist, was shown to induce significant increases in markers of liver injury along with a modest decrease in atherogenic lipids and lipoproteins. To get more insight into whether these effects on liver parameters were compound specific or the effect of mimicking thyrotoxicosis, we studied the effects of supra-physiological levothyroxine dosages on liver parameters, lipids and lipoproteins. We used data of a single-blinded, randomized controlled crossover trial. Herein, healthy volunteers received levothyroxine or no medication for 14 days. Thyroid hormone excess did not induce clinically relevant changes in liver parameters, while significant reductions in total cholesterol, low-density lipoprotein-cholesterol as well as apolipoprotein-B levels were observed in the intervention periods compared with the control periods. Supra-physiological thyroid hormone levels did not induce clinically relevant increases in markers of liver injury after 2 weeks of exposure, while it reduced total cholesterol, low-density lipoprotein cholesterol and apolipoprotein B levels. This suggests that the effects of eprotirome on liver parameters in previous studies were either off-target and compound specific or due to drug-drug interaction at the level of the liver. The results of our study are relevant for the development of novel thyroid hormone agonists to reduce atherogenic lipoproteins.

## Introduction

Since the 1950s, thyroid hormones have been shown to affect lipid homeostasis^1^ and, as such have raised attention as low density lipoprotein cholesterol (LDL-C) lowering agents. Thyroid hormone supplementation results in beneficial effects on lipid and lipoprotein concentrations in patients with hypothyroidism^2^ and to date, several thyroid hormone analogues (e.g., D-thyroxine, tiratricol, and di-iodothyroproprionic acid) have been investigated as lipid-lowering treatments^3–5^. However, none of the thyroid-hormone agonists has successfully completed stage III of clinical development due to cardiac and bone related side effects, including tachycardia^6^ and increased serum osteocalcin^4^, consistent with increased bone turnover^3^.

The cardiac and bone related side effects associated with these agents have, however, focused interest on the development of thyroid hormone agonists with increased liver specificity to avoid deleterious off-target effects and retain beneficial effects on lipid metabolism. Recently, the liver specific thyroid hormone receptor β (TRβ) agonist eprotirome, however, did show cartilage damage in canines^7^, which resulted in the premature termination of the clinical trials conducted to investigate the effects of eprotirome. In addition, in the *AKKA* study, it was shown that liver enzymes increased at 6 weeks in patients with familial hypercholesterolemia (FH) randomized to a daily dose of 50 or 100 µg of eprotirome, while the efficacy of eprotirome could be considered relatively limited with a decrease in LDL-C of 12 and 22% in the low and high dose groups, respectively^8^. The increases in markers of liver injury were already pronounced after 2 weeks of treatment with eprotirome and this led to discontinuation of the study medication in a few patients already before 6 weeks of treatment^8^. It is unknown whether the effects of eprotirome on liver parameters were compound specific or due to a thyrotoxic effect.

We used data of a randomized controlled crossover trial (*OTIHS*) that was performed in 2008/2009^9^, long before the *AKKA* study was enrolling patients. This study was initially performed to assess whether the use of supra-physiological dosages of levothyroxine leads to coagulation activation and inhibition of fibrinolysis and gave us the opportunity to evaluate the effects of supra-physiological levothyroxine dosages on liver parameters, lipids and lipoproteins.

## Materials and Methods

### Study Design and Participants

**Figure 1:**
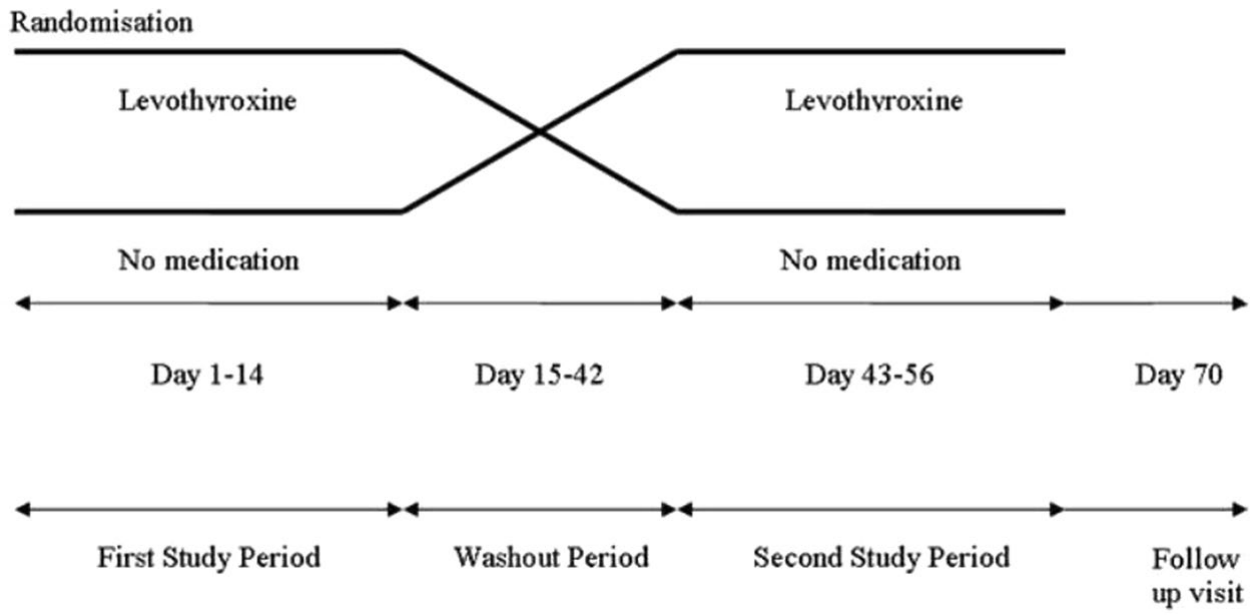
Study Design *OTIHS* Study.

The design, randomisation procedures, and allocation concealment of *OTIHS* were previously described^9^. In summary, *OTIHS* was a single-blinded, randomized, controlled clinical trial with a cross-over design. Healthy volunteers received levothyroxine (intervention period) or no medication (control period) for 14 days, with a wash-out of at least 28 days between the study periods. The order of the two periods was determined by randomisation (Fig. 1). For safety reasons, the study was conducted in two parts. First, participants received a daily dose of 0.3 mg levothyroxine (*Study A; n* = *16*). Only after this dose had proven to be safe, the second part of the study was performed. Herein, participants were using either a daily levothyroxine dose of 0.45 mg (if body weight <80 kg; n = 9) or a daily levothyroxine dose of 0.6 mg (if body weight ≥ 80 kg; n = 3) for 2 weeks (*Study B; n* = *12)*. Safety was monitored as follows throughout both studies: an independent physician collected anthropometric parameters and recorded in a standardized fashion whether any signs or symptoms associated with thyroid hormone excess had occurred. An electrocardiography (ECG) was performed to check for any cardiac arrhythmias. As described previously, slightly increased heart rates were observed after levothyroxine exposure. Moreover, two participants experienced palpitations^9^.

The levothyroxine dosages as used in this study have previously been shown to be safe in healthy volunteers^10^.

Healthy volunteers aged 18 to 40 years were recruited by local advertisements. Volunteers were excluded if they had any of the following: history of thyroid disease or venous thrombosis, any ongoing medical disease or medication use that could affect the primary outcome of the original study^9^. Potentially eligible participants were also excluded in case of any significant abnormalities (e.g. in blood count, inflammation parameters, renal, hepatic, and/or thyroid function tests) at laboratory screening^9^. All participants included in the original study were included in this post-hoc analysis.

The study protocol was approved by the institutional medical ethics committee. The study was done according to the guidelines of the Declaration of Helsinki and the guidelines of Good Clinical Practice. All participants provided written informed consent.

### Laboratory Assessments

Blood samples were collected after an overnight fast. Liver parameters were measured in badge after blood sample storage at −80 °C. Thyroid stimulating hormone (TSH), free thyroxin (FT_4_), tri-iodothyronine (T_3_) and lipid and lipoprotein levels were measured immediately after blood withdrawal.

Aspartate aminotransferase (AST; reference for normal values: <40 U/L), alanine aminotransferase (ALT; reference male: <45 U/L, female: <34 U/L), alkaline phosphatase (ALP; reference: 40–120 U/L), gamma-glutamyltranspeptidase (gamma-GT; reference male: <60 U/L, female: <40 U/L), total and direct bilirubin levels (reference: <17 μmol/L, and <7 μmol/L, respectively) were measured by enzymatic spectrophotometric assays (Roche Diagnostics, Mannheim, Germany).

TSH (reference values: 0.35–4.7 mIU/L), FT_4_ (reference values: 10–23 pmol/L) and T_3_ (reference values: 1.2–2.8 pmol/L) levels were measured by an immune-chemiluminicence assay (ADVIA Centaur immunoassay system; Siemens Healthcare Diagnostics, Marburg, Germany). Total cholesterol (TC; reference: <50 mmol/l), high density lipoprotein (HDL-C; reference: 07–19 mmol/L) and triglyceride (TG; reference: 0.6–2.8 mmol/L) levels were measured by enzymatic colorimetric assays (Beckman Coulter Inc., Mervue, Galway, Ireland). LDL-C levels were calculated using the Friedewald formula^11^. Apolipoprotein B (apoB; reference <1.6 g/L), apolipoprotein AI (ApoA1; reference male: 0.90–1.80 g/L, female: 1.00–2.00 g/L) and lipoprotein(a) (Lp(a) levels; reference: <0,30 g/L) were measured by a nephelometric assay (Beckman Coulter Inc., Mervue, Galway, Ireland).

### Statistical analysis

Statistical analysis was performed using SPSS software, version 20.0 (SPSS Inc, Chicago, IL, USA). The characteristics of the participants are reported as means ± SD or 95% confidence intervals (CI) or median and inter quartile ranges [IQR], where appropriate. Paired samples t-tests and Wilcoxon Signed Rank-tests were used where appropriate. A *P*-value < 0.05 was considered as statistically significant. Relative changes between baseline and follow-up were compared between the intervention and the control period. Study A and B were analyzed separately.

The datasets generated during and/or analyzed during the current study are available from the corresponding author on reasonable request.

## Results

The baseline characteristics of all individuals participating in Study A and Study B are shown in Table 1.

**Table 1:** Participant Characteristics at study entry.

	Study A (n = 16)	Study B (n = 12)
Male, n (%)	9 (56.3)	6 (50.0)
Age, years, mean (range)	30.2 (25–39)	29.3 (26–40)
AST (U/L)	24 ± 3	20 ± 4
ALT (U/L)	25 ± 14	18 ± 4
ALP (U/L)	64 ± 19	65 ± 15
Gamma-GT (U/L)	19 [14–29]	16 [13–19]
Total Bilirubin (µmol/L)	9 ± 4	12 ± 8
Conjugated Bilirubin (µmol/L)	3.7 ± 1.3	4.3 ± 2.1
Total Cholesterol (mmol/L)	4.9 ± 0.9	4.3 ± 0.5
LDL-Cholesterol (mmol/L)	3.1 ± 0.8	2.8 ± 0.5
HDL-Cholesterol (mmol/L)	1.4 ± 0.4	1.2 ± 0.3
Triglycerides (mmol/L)	0.82 [0.54–1.14]	0.83 [0.73–1.14]
Apolipoprotein B (g/L)	0.88 ± 0.22	0.88 ± 0.12
Apolipoprotein A1 (g/L)	1.49 ± 0.24	1.39 ± 0.13
Lipoprotein (a) (g/L)	0.07 [0.02–0.21]	0.08 [0.04–0.33]

Data represent means ± standard deviation or medians [inter quartile range]. Abbreviations: AST = aspartate aminotransferase, ALT = alanine aminotransferase, ALP = alkaline phosphatase, LDL-cholesterol = low-density lipoprotein-cholesterol, HDL-cholesterol = high density lipoprotein-cholesterol, Gamma-GT = gamma-glutamyltranspeptidase, n = number of individual.

### Thyroid function

T_3_ levels increased from (mean ± SD) 1.99 ± 0.31 to 2.61 ± 0.49 nmol/L and from 2.3 ± 0.36 to 3.8 ± 0.49 nmol/L after the intervention periods of Study A and B, respectively (*P* < 0.01 for both compared with the change in the control period).

Mean FT_4_ levels increased from 16.7 ± 3.28 to 28.1 ± 8.1 and from 14.8 ± 1.5 to 41.5 ± 7.2 pmol/L in the intervention periods of Study A and B, respectively (*P* < 0.01 for both compared with the change in the control period). Mean TSH levels decreased from 1.98 ± 0.89 to 0.15 ± 0.17 mIU/L and from 2.49 ± 1.44 to 0.03 ± 0.01 mIU/L in the intervention periods of Study A and B, respectively (*P* < 0.01 for both compared with the change in the control period). A more detailed description of changes in thyroid hormone levels has previously been published^9^.

### Liver Parameters

No differences in baseline levels of liver parameters were observed between study periods, except for ALT levels in Study B which were slightly higher at baseline of the control period compared to baseline levels of the intervention period (19 vs. 16 U/L, respectively (*P* = 0.023). In Study A and B, the intervention period did not yield any significant changes in AST, ALT, ALP or total bilirubin levels compared to the control period. Although we did not observe any significant differences in gamma-GT and conjugated bilirubin levels between study periods of Study A (change of 0% (−1 to 2) in gamma-GT and 0% (−17 to 18) in conjugated bilirubin after the control period, compared to an increase of 4% (−2 to 11) and 10% (−7 to 27), respectively, after the intervention period (*P* = 0.776 and 0.450)), significant differences between study periods were observed in Study B (Table 2). Gamma-GT namely, increased by 7% (−3 to 17) after the intervention period, compared to 1% (0 to 3) after the control period (*P* = 0.033) and conjugated bilirubin increased by 14% (−4 to 32%) after the intervention period compared to a decrease of 8% (−19 to 4) after the control period (*P* = 0.039). None of the liver parameters in either study reached levels above 2 times the normal range.

**Table 2:** Changes in Liver Parameters in the *OTIHS* Study.

	*Study A* (n = 16)				*Study B* (n = 12)			
	Baseline	Week 2	Change from Baseline (%)	*P value* ^†^	Baseline	Week 2	Change from baseline (%)	*P value* ^†^
**AST (U/L)**								
Control period	24 ± 4	23 ± 6	−5 (−14 to 5)	0.775	21 ± 6	22 ± 5	8 (−9 to 25)	0.900
Intervention period	24 ± 4	22 ± 3	−6 (−17 to 4)		20 ± 4	22 ± 6	9 (−11 to 30)	
**ALT (U/L)**								
Control period	26 ± 13	27 ± 18	−2 (−13 to 10)	0.420	19 ± 7	17 ± 4	−7 (−19 to 5)	0.114
Intervention period	25 ± 15	26 ± 14	5 (−7 to 17)		16 ± 4	16 ± 4	4 (−11 to 20)	
**ALP (U/L)**								
Control period	66 ± 17	68 ± 20	3 (−1 to 8)	0.435	65 ± 14	65 ± 15	0 (−3 to 3)	0.976
Intervention period	64 ± 17	65 ± 19	1 (−4 to 6)		66 ± 15	66 ± 14	0 (−5 to 5)	
**Gamma-GT (U/L)**								
Control period	18 [14–29]	20 [14–34]	0 (−1 to 2)	0.776	16 [12–19]	16 [11–18]	1 (0 to 3)	0.033
Intervention period	19 [13–35]	19 [14–37]	4 (−2 to 11)		16 [13–18]	17 [12–18]	7 (−3 to 17)	
**Total Bilirubin (µmol/L)**								
Control period	10 ± 6	9 ± 4	7 (−19 to 33)	0.955	11 ± 5	10 ± 6	−9 (−24 to 7)	0.126
Intervention period	10 ± 5	10 ± 5	8 (−13 to 29)		13 ± 8	15 ± 11	12 (−11 to 35)	
**Conjugated Bilirubin (µmol/L)**								
Control period	3.8 ± 1.9	3.5 ± 1.2	0 (−17 to 18)	0.450	4.2 ± 1.3	3.9 ± 1.6	−8 (−19 to 4)	0.039
Intervention period	3.7 ± 1.4	3.9 ± 1.2	10 (−7 to 27)		4.6 ± 2.2	5.3 ± 2.6	14 (−4 to 32)	

Abbreviations: ALT = alanine aminotransferase; AST = aspartate aminotransferase; ALP = alkaline phosphatase; Gamma-GT = gamma-glutamyl transpeptidase. ^†^ *P* value for difference in relative change between baseline and follow-up in control period vs. intervention period.

### Lipids and Lipoproteins

No differences in baseline levels of lipids and lipoproteins were observed between study periods. Levothyroxine induced significant reductions in TC, LDL-C, and apoB compared with the control period (Table 3). TC levels decreased by 11% (95% CI: 16 to 6%) and by 15% (19 to 11%) after the intervention periods of Study A and B, respectively, compared with an increase by 6% (−2 to +13%) and a decrease by 4% (−8 to −1%), respectively after the control periods (*P* < 0.001 and *P* = 0.007 for differences between study period in Study A and B, respectively).

**Table 3:** Changes in Lipid and Lipoprotein in the *OTIHS* Study.

	*Study A* (n = 16)				*Study B* (n = 12)			
	Baseline	Week 2	Change from Baseline (%)	*P value* ^‡^	Baseline	Week 2	Change from baseline (%)	*P value* ^‡^
**TC (mmol/L)**								
Control period	4.9 ± 0.9	5.1 ± 0.9	6 (−2 to 13)	<0.001	4.6 ± 0.5	4.3 ± 0.5	−4 (−8 to −1)	0.007
Intervention period	5.0 ± 0.9	4.4 ± 0.8	−11 (−16 to −6)		4.4 ± 0.5	3.7 ± 0.3	−15 (−19 to −11)	
**LDL-C (mmol/L)**								
Control period	3.1 ± 0.8	3.2 ± 0.8	6 (−2 to 15)	<0.001	3.0 ± 0.5	2.8 ± 0.5	−5 (−10 to 0)	0.025
Intervention period	3.1 ± 0.9	2.7 ± 0.7	−13 (−19 to −7)		2.9 ± 0.4	2.4 ± 0.3	−17 (−23 to −11)	
**HDL-C (mmol/L)**								
Control period	1.3 ± 0.4	1.4 ± 0.4	6 (−3 to 15)	0.008	1.2 ± 0.4	1.1 ± 0.3	−1 (−7 to 6)	0.037
Intervention period	1.4 ± 0.5	1.3 ± 0.3	−9 (−14 to −3)		1.1 ± 0.2	1.0 ± 0.3	−13 (−21 to −6)	
**Triglycerides (mmol/L)**								
Control period	0.78 [0.50–1.05]	0.75 [0.57–0.98]	9 (−7 to 26)	0.885	0.83 [0.65–0.98]	0.75 [0.63–0.91]	0 (−21 to 20)	0.730
Intervention period	0.89 [0.54–1.20]	0.73 [0.61–1.46]	7 (−11 to 26)		0.82 [0.72–1.12]	0.83 [0.71–0.98]	−4 (−12 to 4)	
**Apolipoprotein B (g/L)**								
Control period	0.88 ± 0.23	0.91 ± 0.24	4 (−2 to 10)	0.001	0.89 ± 0.11	0.83 ± 0.11	−7 (−10 to −4)	0.015
Intervention period	0.88 ± 0.25	0.81 ± 0.22	−8 (−14 to −2)		0.85 ± 0.11	0.71 ± 0.08	−16 (−21 to −12)	
**Apolipoprotein A1 (g/L)**								
Control period	1.45 ± 0.22	1.51 ± 0.25	5 (−2 to + 11)	0.029	1.41 ± 0.15	1.37 ± 0.16	−3 (−5 to 0.4)	0.012
Intervention period	1.52 ± 0.26	1.45 ± 0.20	−5 (−9 to −0.4)		1.37 ± 0.12	1.26 ± 0.12	−8 (−11 to −4)	
**Lipoprotein(a) (g/L)**								
Control period	0.07 [0.02–0.15]	0.08 [0.02–0.24]	18 (1 to 34)	0.027	0.08 [0.04–0.33]	0.07 [0.04–0.38]	−4 (−16 to 8)	0.937
Intervention period	0.07 [0.03–0.21]	0.07 [0.02–0.16]	−5 (−16 to 7)		0.11 [0.03–0.38]	0.11 [0.03–0.45]	−5 (−15 to 6)	

Abbreviations: TC = Total Cholesterol, LDL-C = Low Density Lipoprotein-Cholesterol, HDL-C = High Density Lipoprotein-Cholesterol, TG = Triglycerides ^‡^ *P value* for difference in relative change between baseline and follow-up in control period vs. intervention period.

LDL-C levels decreased by 13% (14 to 3%) and by 17% (23 to 11%) after the intervention periods, respectively, compared with an increase of 6% (−2 to +13%) and a decrease of 5% (−10 to 0%), after the control period (*P* < 0.001 and *P* < 0.025 for between period comparisons in Study A and B, respectively). In both studies, reductions in apoB levels were slightly lower compared with reductions in LDL-C levels. In Study A, apoB levels decreased by 8% (14 to 2%) and by 16% (21 to 12%) after the intervention periods in Study A and B, respectively while a change of +4% (−2 to +10%) and −7 (−10 to −4) were observed when participants did not receive any medication (*P* < 0.001 and *P* = 0.015 for between period differences in Study A and B, respectively).

TG and Lp(a) levels did not significantly change after the intervention period in both studies (Table 3), although we observed a significant difference in Lp(a) levels between the study periods in Study A. In both studies, HDL-C and apoA1 levels decreased after the intervention period while HDL-C and apoA1 levels remained stable during the control period. All relative changes in lipid and lipoprotein levels are reported in Table 3.

## Discussion

We studied the effects of supra-physiological levothyroxine dosages on liver parameters, lipids and lipoproteins in healthy volunteers. Levothyroxine administration for 14 days had no clinically relevant effects on liver parameters, while it resulted in significant reductions in TC, LDL-C, apoB and HDL-C levels. Although differences between study periods were observed in gamma-GT and conjugated bilirubin levels in Study B, these effects were likely partly attributable to normal variation since all gamma-GT levels remained within the reference values. Moreover, the maximal absolute change from baseline was 5% after the intervention period compared with a maximal absolute change from baseline of 9% after the control period. In the *AKKA* stud*y*, conversely, 46% of patients treated with 50 µg of eprotirome had increased gamma-GT levels above the upper limit of normal (ULN), after two weeks of follow-up^8^. All absolute values for conjugated bilirubin levels at follow-up were below the ULN except for one person, whose value was already above ULN at the start of the intervention period.

Our study confirms the potential of thyroid hormone as a lipid-lowering agent, which has been observed in previous studies^1,2^. Levothyroxine led to a decrease in LDL-C of 13% in Study A, and 17% in study B. Considering the fact that this study was performed in healthy volunteers with lower baseline lipid levels, its lipid-lowering potential is definitely comparable to at least the lower dose of eprotirome in the *AKKA* trial (50 µg per day), which showed a decrease of 9% and 13% after treatment with 50 µg and 100 µg per day for 2 weeks, respectively in patients with FH. It is commonly appreciated that the beneficial effects of thyroid hormone on lipids and lipoproteins are primarily caused by stimulation of the TRβ in the liver^12^. The main mechanisms for the effects on lipids and lipoproteins after activation of the TRβ are increasing the expression of the LDL-receptor (LDLR) and promoting 7α-hydroxylase-mediated cholesterol and bile acid secretion^13^. Indeed, liver specific thyroid hormone analogues, with a higher affinity for TRβ over TRα, resulted in a beneficial effect on lipid parameters in pre-clinical and clinical studies^8,14,15^. Unfortunately, the liver specific thyroid hormone analogue eprotirome failed due to cartilage damage in canines^7^. In the *AKKA* study, statistically significant increases in aspartate aminotransferase (AST; *P* < 0.0001), alanine aminotransferase (ALT; *P* < 0.0001), conjugated bilirubin (*P* = 0.0006), and gamma-GT (*P* < 0.0001) were shown in both eprotirome groups compared with placebo^8^. Four patients had to discontinue or interrupt study medication before trial termination due to severe increases in AST and ALT levels. In these four patients, AST concentrations increased to between ULN and six times ULN. ALT concentrations increased to between three and seven times ULN. In contrast, only one patient withdrew because of abnormal liver function tests in a phase II clinical trial by Ladenson and co-workers^14^. For the future of liver specific thyroid hormone analogues it is important to determine whether these adverse effects were caused by a thyrotoxic effect in the liver or whether the effects were off-target and compound specific.

In the present study, thyroid hormone excess did not clinically significantly affect AST, ALT, ALP or total bilirubin levels, in both dosages of levothyroxine. Although the between period differences for gamma-GT and conjugated bilirubin levels were significant in Study B, these effects were possibly attributable to normal variation and, regarding gamma-GT, negligible when compared to the marked increases in a large amount of the patients treated with eprotirome for 2 weeks. AST levels did not increase to a level above ULN (40 U/L) in any participant using levothyroxine, while in the *AKKA* study 67% of patients had AST levels above ULN after 2 weeks of treatment with 50 µg eprotirome (vs. 13% at baseline) and 50% of patients had AST levels above ULN after 2 weeks of treatment with 100 µg eprotirome (vs. 9% at baseline)^8^. Moreover, none of the participants in the present study developed ALT levels above 2 times ULN (ULN: 45 U/L for men and 34 U/L for women) after the intervention period while 29% of patients treated with 50 µg eprotirome and 27% of patients treated with 100 µg eprotirome had increased ALT levels above 2 times ULN after 2 weeks of treatment, in the *AKKA* study (vs. none at baseline for both study periods)^8^. Although liver enzyme abnormalities in patients with thyrotoxicosis due to e.g. Graves’ disease, are frequently observed^16–18^, the results of our study suggest that exogenous thyroid hormone excess does not result in significant effects on markers of liver injury in healthy individuals exposed to levothyroxine for 2 weeks. By studying the effects of levothyroxine exposure on liver parameters in healthy volunteers, an effect of concomitant illness and/or auto-immunity on markers of liver injury is ruled out. Beside this, it is suggested that the frequent liver enzyme abnormalities observed in patients with thyreotoxicosis are, at least partly, caused by therapies such as propylthiouracil^17^. The rapid increase in AST and ALT levels in the *AKKA* study, already present after 2 weeks of follow-up^8^, may therefore well be the result of eprotirome itself or a synergistic effect of statins/ezetimibe and eprotirome via an unknown pathophysiological mechanism that results in increased markers of liver injury. The latter might also be a specific effect of liver-targeted thyroid hormone receptor agonists. This can be illustrated by the fact that VK2809 (formerly MB07811), a liver-selective cytochrome P450-activated prodrug that undergoes first-pass hepatic extraction, also showed mild increases in liver enzymes after 2 weeks of treatment^19,20^. The observation that treatment with MGL-3196, a thyroid hormone agonist with a 28-fold TRβ selectivity over TRα^21^, did not result in increased liver parameters in healthy volunteers with mildly elevated LDL-C levels after 2 weeks, may, however, suggest that it might be possible to develop thyroid hormone receptor agonists that have beneficial lipid lowering effects without adverse effects on liver enzymes^22^. Whether this indeed holds true for the use of MGL-3196 as add-on therapy to a statin with or without ezetimibe in patients with heterozygous familial hypercholesterolemia, is currently being studied (NCT03038022).

Several limitations of our study should be taken into account while interpreting the data. First, we performed a post-hoc analysis and therefore, the study population was not powered for the analyses we performed in this study. Moreover, we performed our analysis in a small study population. Therefore, we cannot exclude the possibility that small or less frequent effects on liver parameters were missed. Since the marked effect of eprotirome on liver parameters was already present in relatively small study groups (n = 24 in 50 µg and n = 22 in the 100 µg arm^8^) it is, however, unlikely that a missed effect in our study would reach the significance as seen in the *AKKA* study. It is of note that in the first study investigating the effects of eprotirome in human Berkenstam and co-workers already observed mild increases in liver enzymes in patients with mild dyslipidemia, after 2 weeks (placebo, 50, 100 or 200 µg; n = 6 per treatment)^23^. Third, since in the current study levothyroxine was only used for 2 weeks, we can also not exclude the possibility that supra-physiological levothyroxine dosages might induce more pronounced liver test abnormalities after longer exposure. However, the increases in markers of liver injury were already pronounced after 2 weeks of treatment with eprotirome (see supplementary data^8,23^). Last, the best approach to investigate whether the effects of eprotirome on liver parameters were compound specific or due to a thyrotoxic effect would obviously be a randomized clinical trial including a direct comparison between eprotirome, levothyroxine and placebo. This is, however, not feasible since eprotirome will no longer be tested in humans. Therefore, our best available approach to address our research question was using the data of the *OTIHS* study which gave us the opportunity to rule out an effect of 2 weeks of exposure with supra-physiological dosages of levothyroxine on liver parameters in healthy volunteers. Since the *AKKA* study was performed in patients with familial hypercholesterolemia, the question remains, however, whether the absence of clinically relevant liver enzyme increases can be observed after exposure with similar levothyroxine dosages, in these patients as well.

In conclusion, supra-physiological thyroid hormone levels induced by supra-physiological dosages of levothyroxine for 14 days did not result in clinically relevant increases in liver parameters, while it reduced TC, LDL-C and apoB levels. Although it is, e.g. due to differences in study design and study population, not possible to directly compare the results of the *AKKA* study with the current study, this might suggest that the effects of eprotirome on liver parameters as previously reported were either off-target and compound specific or due to drug-drug interaction at the level of the liver. The results of this study might be relevant in light of the development of novel thyroid hormone agonists with the purpose to reduce atherogenic lipoprotein particles in the future.

## References

[1] Diekman T, Lansberg PJ, Kastelein JJ, Wiersinga WM (1995). Prevalence and correction of hypothyroidism in a large cohort of patients referred for dyslipidemia. Arch.Intern.Med..

[2] Tzotzas T, Krassas GE, Konstantinidis T, Bougoulia M (2000). Changes in lipoprotein(a) levels in overt and subclinical hypothyroidism before and during treatment. Thyroid.

[3] Ladenson PW (2010). Effects of the thyromimetic agent diiodothyropropionic acid on body weight, body mass index, and serum lipoproteins: a pilot prospective, randomized, controlled study. J.Clin.Endocrinol.Metab.

[4] Sherman SI, Ladenson PW (1992). Organ-specific effects of tiratricol: a thyroid hormone analog with hepatic, not pituitary, superagonist effects. J.Clin.Endocrinol.Metab.

[5] Stamler J (1977). The coronary drug project–findings with regard to estrogen, dextrothyroxine, clofibrate and niacin. Adv.Exp.Med.Biol..

[6] Tancevski I, Eller P, Patsch JR, Ritsch A (2009). The resurgence of thyromimetics as lipid-modifying agents. Curr Opin Investig Drugs.

[7] Karo Bio, 2012. Press Release. Available at http://karopharma.se/en/karo-bio-terminates-theeprotirome-program/ Accessed 16 October 2017.

[8] Sjouke B (2014). Eprotirome in patients with familial hypercholesterolaemia (the AKKA trial): a randomised, double-blind, placebo-controlled phase 3 study. Lancet Diabetes Endocrinol.

[9] Van Zaane B (2011). Alterations in coagulation and fibrinolysis after levothyroxine exposure in healthy volunteers: a controlled randomized crossover study. J Thromb Haemost.

[10] Rogers JS, Shane SR (1983). Factor VIII activity in normal volunteers receiving oral thyroid hormone. J Lab Clin Med.

[11] Fukuyama N (2008). Validation of the Friedewald Equation for Evaluation of Plasma LDL-Cholesterol. J Clin Biochem Nutr.

[12] Duntas LH, Brenta G (2012). The effect of thyroid disorders on lipid levels and metabolism. Med Clin North Am.

[13] Stoekenbroek RM, Kastelein JJ, Hovingh GK (2013). Recent failures in antiatherosclerotic drug development: examples from the thyroxin receptor agonist, the secretory phospholipase A2 antagonist, and the acyl coenzyme A: cholesterol acyltransferase inhibitor programs. Curr Opin Lipidol.

[14] Ladenson PW (2010). Use of the thyroid hormone analogue eprotirome in statin-treated dyslipidemia. N.Engl.J.Med..

[15] Lin JZ (2012). Thyroid hormone receptor agonists reduce serum cholesterol independent of the LDL receptor. Endocrinology.

[16] Gurlek A, Cobankara V, Bayraktar M (1996). Changes in liver biochemical tests at diagnosis and after propylthiouracil therapy for hyperthyroidism. Mater Med Pol.

[17] Gurlek A, Cobankara V, Bayraktar M (1997). Liver tests in hyperthyroidism: effect of antithyroid therapy. J. Clin. Gastroenterol..

[18] Kubota S (2008). Serial changes in liver function tests in patients with thyrotoxicosis induced by Graves’ disease and painless thyroiditis. Thyroid.

[19] Erion MD (2007). Targeting thyroid hormone receptor-beta agonists to the liver reduces cholesterol and triglycerides and improves the therapeutic index. Proceedings of the National Academy of Sciences of the United States of America.

[20] Lian B, Hanley R, Schoenfeld S (2016). A phase 1 randomized, double-blind, placebo-controlled, multiple ascending dose study to evaluate safety, tolerability and pharmacokinetics of the liver-selective TR-beta agonist VK2809 (MB07811) in hypercholesterolemic subjects. J Am Coll Cardiol.

[21] Kelly MJ (2014). Discovery of 2-[3,5-dichloro-4-(5-isopropyl-6-oxo-1,6-dihydropyridazin-3-yloxy)phenyl]-3,5-dio xo-2,3,4,5-tetrahydro[1,2,4]triazine-6-carbonitrile (MGL-3196), a Highly Selective Thyroid Hormone Receptor beta agonist in clinical trials for the treatment of dyslipidemia. Journal of medicinal chemistry.

[22] Taub R (2013). Lipid lowering in healthy volunteers treated with multiple doses of MGL-3196, a liver-targeted thyroid hormone receptor-beta agonist. Atherosclerosis.

[23] Berkenstam A (2008). The thyroid hormone mimetic compound KB2115 lowers plasma LDL cholesterol and stimulates bile acid synthesis without cardiac effects in humans. Proc.Natl.Acad.Sci.USA.

